# Specific Inhibition of the VEGFR-3 Tyrosine Kinase by SAR131675 Reduces Peripheral and Tumor Associated Immunosuppressive Myeloid Cells

**DOI:** 10.3390/cancers6010472

**Published:** 2014-02-28

**Authors:** Nicolas Espagnolle, Pauline Barron, Marie Mandron, Isabelle Blanc, Jacques Bonnin, Magali Agnel, Erwan Kerbelec, Jean Pascal Herault, Pierre Savi, Françoise Bono, Antoine Alam

**Affiliations:** 1 UMR5273 INSERM U1031/CNRS/EFS StromaLab, Toulouse 31432, France; E-Mail: nicolas.espagnolle@efs.sante.fr; 2 Sanofi Recherche et Développement, Early to Candidate DPU, Toulouse 31036, France; E-Mails: pauline.barron@sanofi.com (P.B.); marie.mandron@sanofi.com (M.M.); isabelle.blanc@sanofi.com (I.B.); jacques.bonnin@sanofi.com (J.B.); jean-pascal.herault@sanofi.com (J.P.H.); pierre.savi@hotmail.fr (P.S); franç oise.bono@sanofi.com (F.B.); 3 Molecular Biology Unit, Biologics Department, Sanofi, Vitry-sur-Seine 94400, France; E-Mails: magali.agnel@sanofi.com (M.A.); erwan.kerbellec@sanofi.com (E.K.)

**Keywords:** VEGFR-3, macrophages, MDSCs, angiogenesis, lymphangiogenesis

## Abstract

Myeloid derived suppressor cells (MDSCs) and tumor-associated macrophages (TAMs) represent prominent components in cancer progression. We previously showed that inhibition of the VEGFR-3 pathway by SAR131675 leads to reduction of TAM infiltration and tumor growth. Here, we found that treatment with SAR131675 prevents the accumulation of immunosuppressive blood and splenic MDSCs which express VEGFR-3, in 4T1 tumor bearing mice. Moreover we showed that soluble factors secreted by tumor cells promote MDSCs proliferation and differentiation into M2 polarized F4/80+ macrophages. In addition, cell sorting and transcriptomic analysis of tumor infiltrating myeloid cells revealed the presence of a heterogeneous population that could be divided into 3 subpopulations: (i) immature cells with a MDSC phenotype (GR1+/CD11b+/F4/80^−^); (ii) “immuno-incompetent” macrophages (F4/80^high^/CD86^neg^/MHCII^Low^) strongly expressing M2 markers such as Legumain, CD206 and Mgl1/2 and (iii) “immuno-competent”-M1 like macrophages (F4/80^Low^/CD86^+^/MHCII^High^). SAR131675 treatment reduced MDSCs in lymphoid organs as well as F4/80^High^ populations in tumors. Interestingly, in the tumor SAR131675 was able to increase the immunocompetent M1 like population (F4/80^low^). Altogether these results demonstrate that the specific VEGFR-3 inhibitor SAR131675 exerts its anti tumoral activity by acting on different players that orchestrate immunosuppression and cancer progression in a tumoral context: MDSCs in peripheral lymphoid organs and TAMs infiltrating the tumor.

## 1. Introduction

Despite tremendous efforts to harness the power of the immune system, the use of vaccines and other immunotherapies to fight cancer has largely ended in failure. Within the past few years, researchers have identified a heterogeneous myeloid population known as myeloid-derived suppressor cells (MDSCs) in cancer patients [1,2,3] and also in rodent tumor models and suggested that these cell populations may be one of the causes of the failure of immunotherapy. Growing evidence suggests that MDSCs, which have been named “immature myeloid cells” or “myeloid suppressor cells” (MSCs), play a critical role during the progression of cancer. These cells are immature myeloid cells that fail to complete their differentiation and are endowed with a tremendous immunosuppressive potential as a common feature [4,5]. In mice, MDSCs were originally described as a population of GR1+CD11b+ cells [6]. More recently, MDSCs were divided into two subsets based on the presence of the molecules Ly6C and Ly6G: cells with a granulocytic phenotype, positive for the Ly6G marker; and cells with a monocytic phenotype, positive for the Ly6C marker [7,8]. Human MDSCs have less been described and until now no specific marker has been identified. They express common myeloid markers such as CD11b, CD33, and HLA-DR, often in combination with CD14 (for monocyte lineage) or CD15 for granulocytic lineages [3,9]. In cancer, the number of “MDSCs” generally increases in the peripheral blood and in the tumor. A good correlation between MDSC levels and clinical cancer stage and grade has also been described [10,11,12].

Tumor Associated Macrophages (TAMs) represent another immunosuppressive population found in the tumor microenvironment. Macrophages are plastic cells; they can adopt different phenotypes depending on the microenvironmental context which can drive their differentiation either toward a “classic” (M1) or an “alternative” (M2) activation state [13]. It has been suggested that TAMs mainly display an M2-like phenotype [14] and participate in tumor growth by promoting immune suppression, angiogenesis through producing VEGFs and angiopoietin 1/2 [15], and invasion and metastasis by secretion of proteases such as MMP9 and cathepsins. Therefore, various growth factors and chemokines secreted by TAMs, like epidermal growth factor (EGF), transforming growth factor-β (TGF-β), interleukin-8 (IL-8) and tumour necrosis factor-α (TNF-α) contribute to the migration of tumour cells towards vessels and provide proliferative and anti-apoptotic signals to these cells [16,17]. Accumulating evidence has clearly demonstrated, in various murine tumor models and in clinical situations, that a correlation exists between the amount and/or density of TAMs and tumor progression and aggressiveness [18,19].

While both TAMs and MDSCs present immunosuppressive activity in a tumor context, the complementarity and/or relationship between these two populations are not well understood. It has previously been proposed that MDSC entering the tumor microenvironment (TME) could differentiate into TAMs or TANs (tumor-associated neutrophils) in mice after adoptive transfer of freshly isolated GR1+ cells [20,21,22].

In this study we demonstrate that MDSCs are able to proliferate and differentiate into M2 macrophages in response to tumor secreted factors. Interestingly, the previously described VEGFR-3 inhibitor, SAR131675, reduces the number of circulating MDSCs, increases the proportion of tumor associated “M1-like” macrophages and reduces tumor growth and metastasis. This work confirms the previously reported action of SAR131675 on key myeloid protagonists in tumor development and provides a rationale for the use of VEGFR-3 inhibitors in inflammatory solid tumor treatment.

## 2. Results

### 2.1. Effect of SAR131675 on Spleen Weight of 4T1-Bearing Mice

**Figure 1:**
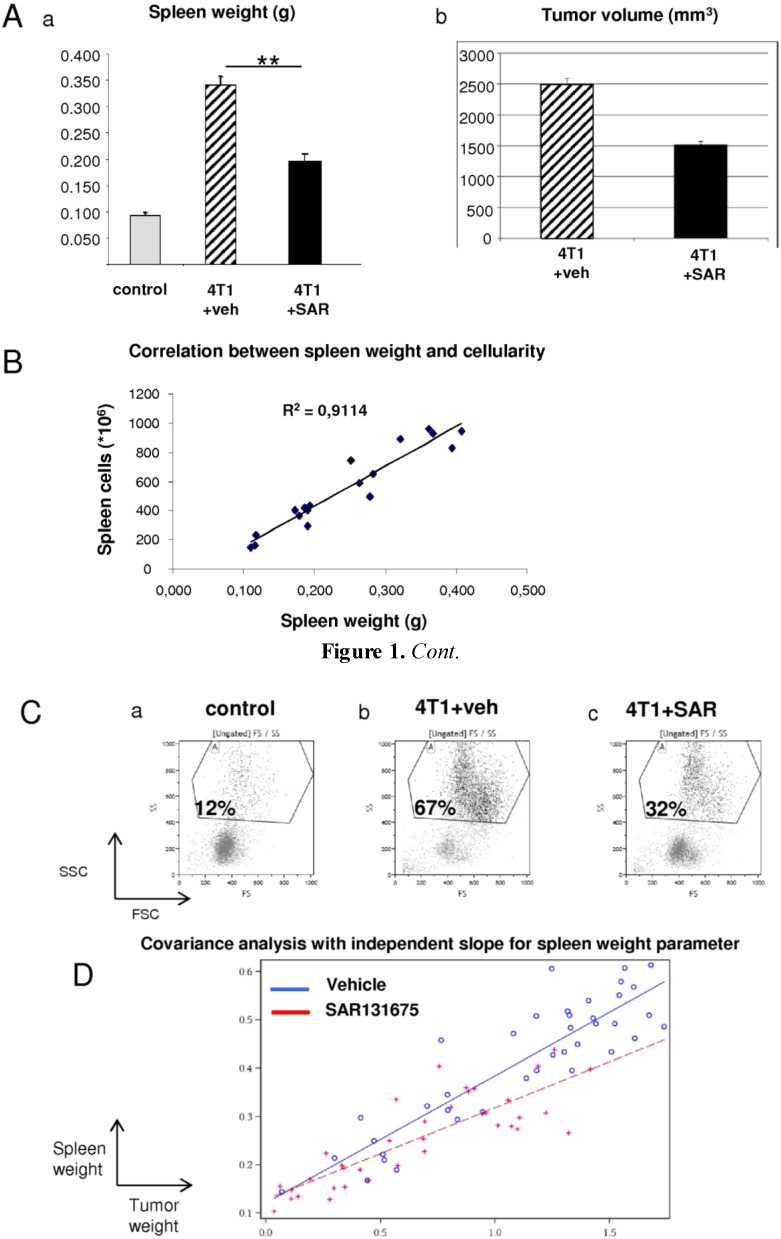
Increase of spleen weight in 4T1 model and effect of SAR131675. (**A**) (**a**) Spleen weight in control mice and in 4T1-bearing mice treated with vehicle (veh: methyl cellulose) or with SAR131675 (SAR) at 100 mg/kg/d. (** *p* ≤ 0.01). (**b**) tumor volume in mice treated with vehicle or with SAR131675 (SAR) at 100 mg/kg/d. (**B**) A regression curve was calculated between spleen weight and cellularity, (R^2^ is mentioned next to the curve). (**C**) Cytometry dot plots showing the side scatter *vs*. forward scatter of splenocytes isolated from control mice (**a**) 4T1-bearing mice (**b**) and 4T1-bearing mice treated with SAR131675 (**c**). (**D**) Analysis of covariance (ANCOVA) of the relation between tumor and spleen weight in vehicle *vs*. SAR131675-treated animals. The data from three experiments were pooled in order to study a greater range of tumor weights (39 animals in the vehicle treated groups and 36 in the SAR treated groups).

In a previous study, we described SAR131675, a specific VEGFR-3 Tyrosine kinase inhibitor, which presents anti-tumoral and anti-metastatic activities in different tumor models in mice and in particular in the mammary 4T1 model [23]. This poorly immunogenic, BALB/c-derived transplantable tumor shares several characteristics with human breast tumors and is an established model for metastatic cancer. This model is characterized by splenomegaly [24]. As shown in Figure 1A, spleen weight from 4T1-bearing mice was 3-fold higher than in control mice. Interestingly, SAR131675 dramatically reduced tumor-induced splenomegaly in 4T1 mice (Figure 1Aa) with inhibition of tumor volume at the same time (Figure 1Ab). Figure 1B shows that splenomegaly was not due to oedema or to matrix synthesis, but to an increase of infiltrating cells. This splenomegaly was correlated with the accumulation of a new cell population within the spleen. These cells are sensitive to 6-thioguanine *ex vivo* demonstrating that this population is not the result of 4T1 metastasis in the spleen [25]. To characterize these spleen invasive cells we performed flow cytometry analysis on total spleen from control and 4T1 tumor-bearing mice. Three weeks after tumor implantation a cell population characterized by Forward light scatter/Side light scatter parameters at high level (FSC^high^/SSC^high^) was present in the spleen from tumor-bearing, but not from control mice (Figure 1C). Moreover, SAR131675 specifically decreased the proportion of this new population (Figure 1C). To determine whether SAR131675 may have a direct effect on splenic cells, we performed an analysis of covariance (ANCOVA) of the relation between tumor and spleen weight in vehicle *vs*. SAR131675-treated animals. The data from three experiments were pooled in order to study a greater range of tumor weights, and the results are shown on Figure 1D. During the ANCOVA, we found that the slope of the regression curve was significantly (*p* < 0.0001) different from 0, confirming that the spleen weight and tumor weight are strongly related. Furthermore, the slope for the vehicle-treated animals was significantly different from the slope for the SAR131675-treated animals (*p* < 0.02), showing that the spleen weight increased less with tumor weight in the SAR131675-treated animals as compared to the vehicle-treated animals. As can be seen in Figure 1D, for an identical tumor weight, the spleen weights were distinctly lower in SAR131675-treated animals. Taken together, these results demonstrate that SAR131675 reduced splenomegaly by specifically inhibiting the accumulation of a new cell population in the spleen.

### 2.2. Effect of SAR131675 on 4T1 Tumor-Induced Splenic and Blood GR1CD11b Cells

**Figure 2:**
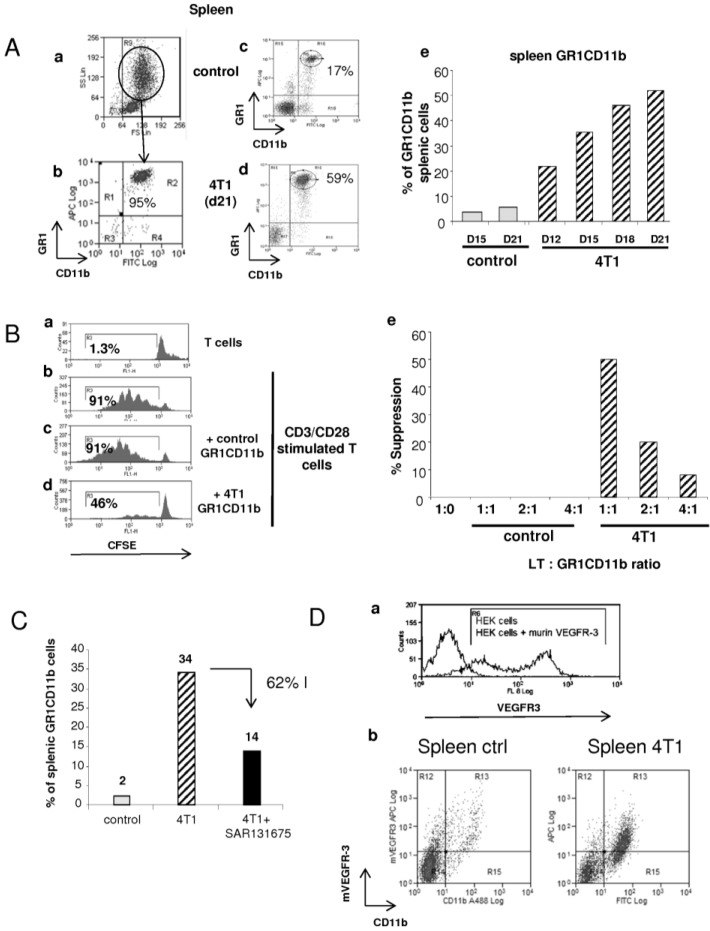
Splenic GR1CD11b cells characterization in the 4T1 model and effect of SAR131675. (**A**) At day 21 after tumor implantation, spleen cells from control and 4T1 mice were stained with fluorochrome-coupled anti-GR1 and anti-CD11b antibodies (**a**–**d**). Kinetics of splenic GR1CD11b cell proportion during tumor growth (**e**). (**B**) CFSE labeled purified CD4+ T cells from control spleen were stimulated (**b**) or not (**a**) by anti CD3/CD28 beads. Activated CD4+ T cells were in co-culture with control (**c**) or 4T1 (**d**) derived GR1CD11b cells. GR1CD11b cells were added at different ratios and their effect was quantified as follow: [1 − (proliferation with 4T1 GR1CD11b cells/proliferation with normal GR1CD11b cells)] × 100 (**e**). (**C**) Effect of SAR131675 on splenic GR1CD11b cells in 4T1 bearing mice. GR1CD11b cells derived from the spleen of 4T1 tumor free-mice were used as control. (**D**) Expression of VEGFR-3 on splenic CD11b cells from control and 4T1 bearing mice. (**a**) validation of the anti-VEGFR-3 antibody on HEK cells transfected with empty plasmid (negative control) or with murine VEGFR-3 encoding plasmid. (**b**) Double staining of splenocytes, derived from control mice or from 4T1 bearing mice, with anti-VEGFR-3 and anti-CD11b antibodies.

In rodents, MDSCs have been characterized by co-expression of two key markers: GR1 and CD11b [6]. If we consider just the new FSChigh/SSChigh cell population described in Figure 1, 95% of this cell population expressed GR1 and CD11b markers (Figure 2Aa,b). As shown in Figure 2A, three weeks after 4T1 cells implantation, the percentage of GR1CD11b cells was highly increased in spleen and in plasma (Figure 2Aa–d) and Supplementary Figure S1Aa–c). If we consider the entire population, the proportion of splenic and blood GR1CD11b cells was about 17% in control mice, whereas this proportion reached 59% in the spleen and 65% in the blood of tumor-bearing mice (Figure 2Ac,d and Figure S1Aa–c). The increase in splenic and blood GR1CD11b cells started early after 4T1 implantation (22% in the spleen and 35% in blood 10–12 days after tumor implantation) and went up gradually until day 21 (50% in the spleen and 70% in blood, Figure 2Ae and Figure S1Ac). While the number of GR1CD11b cells increased dramatically in the spleen and blood from tumor-bearing mice in comparison to control mice, the phenotype of these cells from the two mouse populations was not different. These cells expressed CD115 and IL4R and did not express F4/80, MHC-II and CD86, indicating they were in an immature myeloid state not able to stimulate T cells (Supplementary Figure S1B and Figure S2A).

We therefore asked whether GR1CD11b cells derived from 4T1-bearing mice and control mice were functionally different. For this aim, purified GR1CD11b cells from each population were purified and we investigated their immunosuppressive activity. T cells were stimulated by anti-CD3/CD28 beads in the presence of different ratios of sorted splenic GR1CD11b cells obtained from control or 4T1 bearing mice (Figure 2B). Whereas non-stimulated T cells didn’t proliferate (Figure 2Ba), treatment with anti CD3/CD28 beads strongly induced their proliferation (91% of proliferating T cells, Figure 2Bb).

In the presence of GR1CD11b cells obtained from control mice, T cell proliferation was not altered (91% of proliferating T cells, Figure 2Bc) even at the 1:1 ratio. In contrast, GR1CD11b cells derived from 4T1 bearing mice inhibited T cell proliferation in a dose dependent manner. The maximal inhibition of cycling T cells (46%) was reached at the ratio 1:1 (Figure 2Bd,e).

Under physiological conditions, the ratio between splenic CD4+ T cells and GR1CD11b cells was 4 to 10 (45% CD4+ T cells *vs*. 5%–10% GR1CD11b cells). In mice bearing 4T1 tumors, this ratio was inverted and represented 0.1 to 0.2 (5% of CD4+ T cells and 50% of GR1CD11b cells; Supplementary Figure S1C). Since in our T cell proliferation experiment a ratio of 1:1 was used, these results highlight an impressive immunosuppressive capacity of GR1CD11b cells from 4T1-bearing mouse spleen (Figure 2Be). Interestingly, when mice were treated with SAR131675, there was a clear reduction of the splenic and blood GR1CD11b cell proportion (34% to 14% in spleen Figure 2C and 66% to 36% in blood, Supplementary Figure S1D). Furthermore, a large proportion of CD11b cells expressed VEGFR-3 in 4T1 and in control mice as shown in Figure 2D. These results demonstrate that SAR131675 prevents the increase of blood and splenic myeloid immunosuppressive GR1CD11b cells induced by tumor progression.

### 2.3. Characterization of Tumor Infiltrating Myeloid Subpopulations and Effect of SAR131675

**Figure 3:**
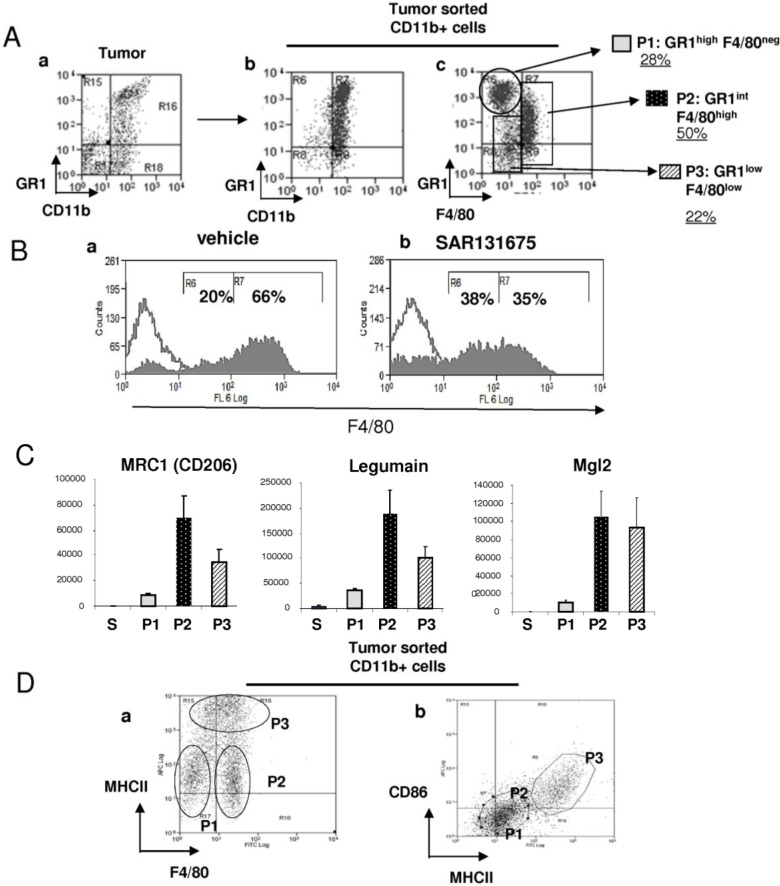
Characterization of 4T1 tumor infiltrating myeloid subpopulations and effect of SAR131675. (**A**) At day 21 after tumor implantation, a single cell suspension was prepared after enzymatic digestion of 4T1 tumors. Flow cytometric analysis was performed on the total cell population (**a**) or after purification of CD11b+ cells (**b**,**c**). Cells were stained with anti GR1, CD11b and F4/80 antibodies. Different gated myeloid subpopulations are noted P1, P2 and P3 (**c**). (**B**) Analysis of the F4/80 expression on CD11b cells sorted from 4T1 tumors from untreated (**a**) or from SAR131675 treated mice (**b**). Histograms are representative of 3 independent experiments. (**C**) Transcriptomic analysis for MRC1, Legumain and Mgl2 from sorted P1 (GR1^High^F4/80^−^ in grey block), P2 (GR1^Int^F4/80^High^ in dark and white points block) and P3 (GR1^Low^F4/80^Low^ in white and dark oblique hatching block) populations. (**D**) CD11b+ cells sorted from 4T1 tumors were stained with fluorochrome-coupled anti-F4/80, anti-MHCII and anti-CD86 antibodies.

After analysis of peripheral myeloid cells in 4T1-bearing mice, we investigated the presence of GR1CD11b cells in the tumor microenvironment. For this purpose, tumors were harvested, dissected and digested by enzymatic treatment, to obtain a single cell suspension. After purification, CD11b+ cells were further stained with anti-GR1 and anti-F4/80 antibodies (Figure 3Aa–c). F4/80 staining revealed the presence of three major populations: P1: corresponding to F4/80-negative immature cells (GR1^high^/CD11b+/F4/80^−^: 28%), P2: (GR1^int^/CD11b+/F4/80^high^: 50%), and P3: GR1^Low^/CD11b+/F4/80^Low^: 22%), (Figure 3Ac). Interestingly, treatment with SAR131675 reduced the number of macrophages expressing high levels of F4/80 (from 65% to 35%) and increased the number of macrophages with lower F4/80 levels (from 20% to 38%; Figure 3Ba,b). The phenotype and biological functions of these three subpopulations were further investigated. Cell sorting and transcriptomic analysis revealed that in contrast to F4/80 negative cells (P1), all F4/80 positive macrophages (P2 and P3) expressed significant levels of MRC1, legumain and Mgl2, demonstrating that P2 and P3 cells represent two distinct subpopulations of macrophages (Figure 3C). To distinguish between these two populations, we stained them with the two immune markers MHCII and CD86, two molecules required for CD4+ T cell activation. On the basis of these stainings, we showed that P2 populations express lower MHCII levels but not CD86 levels, suggesting that they are not endowed with immunostimulatory potential characteristic of the M2 phenotype (Figure 3Da,b). In contrast, the P3 population expressed high levels of MHCII and CD86 (like peritoneal macrophages, data not shown) and lower levels of MRC1 and legumain (M2 markers) suggesting that these macrophages could potentially be M1-like macrophages with immunostimulatory activity (Figure 2Cc,Da,b). Altogether, these findings demonstrate that tumors are infiltrated with heterogeneous macrophage populations and inhibition of the VEGFR-3 pathway by SAR131675 may modify the ratio of M2/M1-like macrophages in the tumor.

### 2.4. 4T1-Derived Factors Induce the Differentiation of GR1CD11b Cells in F4/80+ Macrophages

**Figure 4:**
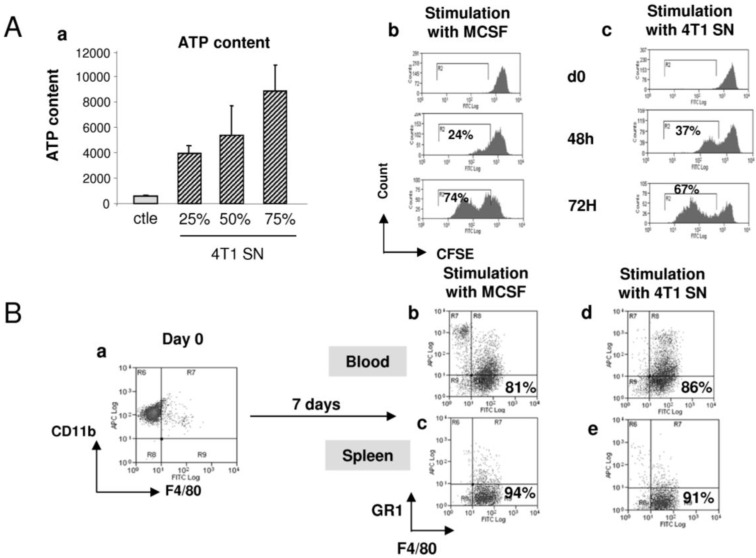
Tumor derived factors induce GR1CD11b cell proliferation and differentiation in tumor associated macrophages. (**A**) CD11b+ cells purified from spleen were incubated with different doses of conditioned medium (25%, 50%, 75% of 4T1 cells culture supernatant). ATP content was evaluated after 72 h of culture (**a**). MCSF (**b**) or 4T1 conditioned medium (**c**)-treated GR1CD11b cells were stained with CFSE, and analyzed after 48 and 72 h as indicated. % of cycling cells is noted. (**B**) Blood (**b**–**d**) or splenic (**c**–**e**) purified GR1CD11b cells (**a**) were incubated during 7 days in the presence of murine recombinant MCSF (100 ng/mL) (**b**,**c**) or 4T1 supernatant conditioned medium (**d**,**e**). Cells were stained with fluorochrome-coupled anti-GR1 and anti-F4/80 antibodies. % of GR1^−cow^/F4/80^+^ differentiated macrophages is noted.

**Figure 5:**
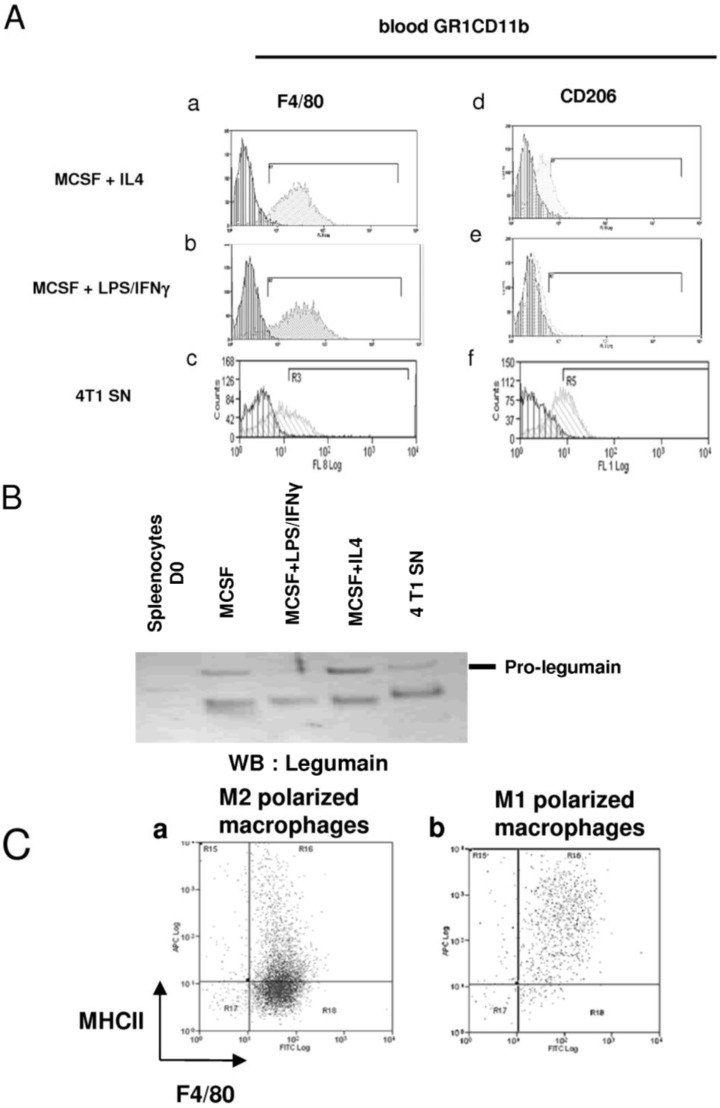
Impact of tumor derived factors on GR1CD11b cell polarization into M1/M2 macrophages. (**A**) Blood purified GR1CD11b cells were incubated with MCSF during 6 days. After differentiation, macrophages were polarized during 24 h with IL4 (**a**–**d**) or LPS/IFNγ (**b**–**e**) or 4T1 conditioned medium (**c**–**f**). Polarized macrophages were stained with fluorochrome-coupled anti-F4/80 (**a**–**c**) or anti-CD206 (**d**–**f**) antibodies. Vertical hatching is used for isotype controls and oblique hatching for staining with the specific markers. (**B**) Western blot analysis showing the expression and processing of Legumain protein in splenocytes and in different subtypes of macrophages described in A. (**C**) M2 (**a**) and M1 (**b**) polarized F4/80+ macrophages were stained with APC coupled anti-MHCII and FITC coupled anti-F4/80 antibodies.

Previous results demonstrated that heterogeneous myeloid cell populations, which contain immature and mature cells, infiltrate tumors. The link between both these myeloid populations is not well established. Since GR1CD11b cells are present in the blood/spleen and in tumors, we hypothesized that this immature population could further differentiate within the tumor microenvironment. In this context, we evaluated the effect of 4T1 cell conditioned medium on GR1CD11b cell behaviour. As indicated in Figure 4A, 4T1 culture-derived soluble factors induced in a dose-dependent manner the proliferation of sorted blood GR1CD11b cells after 72 h of culture as measured by ATP incorporation and by CFSE labelling (Figure 4Aa–c). The same results were obtained with GR1/CD11b cells sorted from the spleen or bone marrow (data not shown). Interestingly, seven days sustained stimulation of blood (and splenic or BM) GR1CD11b F4/80^−^ cells with murine recombinant MCSF (Figure 4Bb,c), or with 4T1-conditioned medium (Figure 4Bd,e) lead to their differentiation into macrophages. This differentiation was evidenced by the loss of the immature marker GR1 and acquisition of the macrophage marker, F4/80. These cells are myeloid derived immature pluripotent cells, which are able to proliferate and differentiate into macrophages under the effect of 4T1-derived soluble factors within the tumor environment. To go further in the characterisation of the phenotype of these differentiated macrophages, sorted GR1CD11b cells were differentiated into macrophages in the presence of MCSF, and then stimulated with IL4 or LPS/IFNγ cocktail to polarize cells into M2 or M1 macrophages, respectively (Figure 5A). The phenotype of these differentiated and well-characterized macrophages was compared to the one obtained after treatment with 4T1 supernatant. Like MCSF and MCSF+IL4 treatments, 4T1 supernatant induced the expression of CD206 and legumain, confirming that 4T1 cells produce factors able to differentiate MDSCs into M2 macrophages (Figure 5Aa–f and 5B). We also found that 4T1 cells produce MCSF and IL13 which may act on MCSFR and IL4 receptors to complete the differentiation into M2 macrophages (Supplementary Figure S2B). In addition, we detected VEGFC in the tumor microenvironment *in vivo* but not in 4T1 cells *in vitro*. These results suggest that the MCSFR and IL4R pathways are important for the proliferation and differentiation of MDSCs *in vitro*. However, it is important to note that SAR131675 does not inhibit the MCSFR-TK (Supplementary Figure S2C) suggesting that *in vivo* other pathways, and mainly the VEGFR-3 pathway, could explain the effects of SAR131675 on MDSCs.

Interestingly, when macrophages were treated with IL4 or MCSF alone, they expressed low levels of MHCII (Figure 5Ca). In contrast, after treatment with LPS/IFNγ these macrophages expressed high levels of MHCII (Figure 5Cb). These two polarized populations are very similar to tumor infiltrating MHCII^high^ and MHCII^low^ myeloid cells described previously (Figure 3). Altogether, these results suggest that “immuno-incompetent” macrophages (GR1^int^/F4/80^high^/CD86^neg^/MHCII^low^) could represent M2 polarized macrophages whereas the “immuno-competent” (GR1^Low^/F4/80^Low^/ CD86^+^/MHCII^High^) homologues may correspond to “immuno-competent” M1 macrophages.

## 3. Discussion

Tumor-infiltrating immune cells are a hallmark of most solid tumors, and the presence of varied immune populations significantly affects clinical outcomes for patients with cancer. Historically, tumor-infiltrating immune cells have been viewed as restraining tumor progression [26], but in recent years, it has become more widely appreciated that chronic immune responses play critical roles in promoting tumor progression. Therefore, understanding the molecular mechanisms by which malignant cells derail antitumor immune responses to favor disease progression is critical. In that context, we have previously shown that SAR131675, a selective VEGFR-3 inhibitor, reduces TAMs infiltration into tumors. In this study, we used an orthotopic and syngenic mammary tumor model to characterize circulating and tumor associated immunosuppressive cells, to establish a relationship between these populations and to investigate the *in vivo* effect of SAR131675 on these different immune cell populations.

We first confirmed that 4T1 bearing mice exhibit significant expansion and accumulation of GR1CD11b cells in spleen and blood as described previously [5,8]. Interestingly, we demonstrated for the first time that tumor-induced accumulation of GR1CD11b cells is dramatically reduced by the blockade of VEGFR-3 signaling by SAR131675. This reduction was also associated with an important decrease of splenomegaly which was strongly correlated with tumor growth. We also showed that this splenomegaly was due to the accumulation of GR1CD11b cells. Interestingly, analysis of covariance showed that the spleen weight increased less with tumor weight in the SAR131675-treated animals as compared to the vehicle-treated animals, suggesting a direct effect of SAR131675 on cells infiltrating the spleen. It is also noteworthy that cytometric and RT-PCR analysis demonstrated the expression of VEGFR-3 in GR1CD11b cells (Figure 2D and data not shown), further suggesting a direct effect of SAR131675 on these cells. It has been already demonstrated that 4T1 tumor-derived GR1CD11b cells contribute to the decrease of immune surveillance [27]. Here we confirmed the functional immunosuppressive effect of these cells, which represents a “trademark” of MDSCs. Indeed, potent inhibition of T cell proliferation by 4T1 tumor-derived GR1CD11b cells was observed with a (1:1) mixture of CD4+ T cells and GR1CD11b cells. This ratio is 10-fold below the physiopathological ratio (1:10), suggesting a strong immunosuppressive potential of 4T1 derived GR1CD11b cells. Interestingly we also observed an increase of GR1CD11b cells in the bone marrow of these mice demonstrating a tumor-induced myelopoiesis in accordance with the work of Shojaei *et al*. (data not shown) [28]. By CFSE labelling or ATP incorporation approaches, we demonstrated that 4T1 soluble factors such as MCSF are able to induce a robust MDSCs proliferation *in vitro*, confirming the precursor status of these cells. These results are strengthened by the work of Movahedi *et al*. who demonstrated the presence of precursors of TAMs (Ly6C^High^) with strong proliferation in the 4T1 model, which continuously renewed all non-proliferating intra-tumor TAM subsets [29].

Cytofluorometric analysis associated with cell sorting and transcriptomic analysis of tumor infiltrating myeloid cells revealed the presence of heterogeneous macrophage subpopulations: (i) A MHCII^Low^/CD86^−C^F4/80^High^ macrophage population expressing MRC1 and legumain corresponding to “M2-like” macrophages without immunostimulatory properties (P2 of Figure 3); (ii) a second macrophage population cells was characterized by a MHCII^High^/CD86^+^/F4/80^Low^ phenotype expressing low levels of MRC1 and legumain, corresponding to “M1-like” macrophages with potential immunostimulatory activity (P3 of Figure 3). These observations are in agreement with those reported in human cancer situations, showing that one of the major features of tumor associated macrophages is the down-regulation of MHC class II molecules [30,31]. These macrophage populations are very plastic and could move from one phenotype to another under the influence of tumoral microenvironment-derived cytokines [17]. Interestingly the amount of “M1-like” macrophages was increased following treatment with SAR131675.

In addition to both these macrophage populations, we noted the presence in the tumoral microenvironment (TME) of a GR1CD11b cell population with an immature status similar to that of the population isolated from the blood and spleen of tumor-bearing mice and characteristic of MDSCs. These cells do not express F4/80 and CD86 and express low levels of MHCII, MRC1, Legumain and Mgl2. It has been shown that MDSCs entering the TME can differentiate into TAMs or TANs in mice [21,32], suggesting that tumor-derived factors induce their functional differentiation into macrophages without immunostimulatory properties, therefore promoting tumor growth. The present data demonstrate that 4T1 tumor-derived factors induce the differentiation of splenic and blood GR1CD11b cells into F4/80+ macrophages. These results confirm that once MDSCs infiltrate the tumor, a small fraction can differentiate into M1-like MHCII^High^ macrophages, but the larger fraction differentiates into M2-like MHCII^Low^ macrophages (summary in schematic drawing, Supplementary Figure S3).

Interestingly, we have previously shown that SAR131675 reduces TAM infiltration in the 4T1 and Rip1Tag2 models. In this report, we showed that SAR131675 reduces the circulating and spleen GR1CD11b immunosuppressive cells. This immunomodulatory activity of VEGFR-3 TK inhibitor could lead to the restoration of immunostimulation of T cells within the tumor, therefore participating into inhibition of tumor growth. In addition we showed that in contrast to sorafenib or sunitinib, this compound does not have any inhibitory effect on T cell proliferation [33,34]. These results highlight the importance of targeted therapy with specific kinase inhibitors. Indeed although multikinase inhibitors possess VEGFR-3 inhibitory activity, they also have immunosuppressive potential through inhibition of T cell responses and by modulating antigen presenting cells and their precursors. For example, imatinib and sunitinib induce secretion of anti-inflammatory IL-10 in macrophage cultures, indicating that treatment with these inhibitors might contribute to an immunosuppressive microenvironment in GIST [35]. In addition, it has been reported that sorafenib induces a significant increase of tumoral infiltration of F4/80+ cells. Depletion of macrophages in combination with sorafenib significantly inhibited tumor progression, tumor angiogenesis, and lung metastasis compared with mice treated with sorafenib alone [36] further demonstrating that multikinase inhibitors are endowed with immunosuppressive activities.

Concerning the specific immunomodulatory role of VEGFR3, it has been reported that VEGFR-3 is expressed on macrophages in several human pathological contexts like brain ischemia, kidney transplantation, chronic airway inflammation, corneal injury [37,38,39,40,41] and cancers [42,43]. It has also been demonstrated that VEGF-C promotes immune tolerance in murine melanoma through deletion of melanoma specific CD8+ T cells. These data are also in agreement with recent work showing that VEGF-C and VEGF-D blockade inhibits inflammatory skin carcinogenesis [44]. These data further suggest that specific inhibition of the VEGFR-3 axis leads to the reduction of myeloid immunosuppressive cells *in vivo* and improvement of the immune response. Circulating bone marrow-derived cells expressing VEGFR-3 were significantly increased in Small Cell Lung Cancer patients, and were associated with lymph node metastasis [45]. These cells were described as lymphatic/vascular endothelial progenitor cells, but their putative immunosuppressive activities and their potential to differentiate into macrophages are still to be demonstrated. Since myeloid cells are highly sensitive to pathogen associated molecular patterns we are working on the production of an ultra-pure VEGFC preparation to address this important question. We conclude that, in addition to its role on lymphatic and endothelial vasculature, the specific VEGFR-3 inhibitor SAR131675 has an anti tumor effect by acting on different mechanisms through which key players of the inflammatory response orchestrate tumor progress, confirming that VEGFR-3 axis is an interesting therapeutic target for cancer treatment. Moreover these data highlight the fact that the specific inhibition of VEGFR-3 tyrosine kinase is more suitable than the blockade of multikinase pathways pointing out the interest of a new class of specific kinase inhibitors for cancer treatment.

## 4. Experimental

### 4.1. Reagents, Cell Line and Tumor Model

SAR131675, 4T1 cells and orthotopic tumor model development have been described previously [23].

### 4.2. Isolation of GR1CD11b Cells and TAMs

Tumors and spleen were harvested, dissected, and digested by enzymatic treatment to obtain single cell suspensions. To purify circulating, splenic, bone marrow and tumor-infiltrating myeloid cells, CD11b+ cells were positively selected with anti-CD11b microbeads using LS columns following the manufacturer’s instructions (Miltenyi Biotec, Bergisch Gladbach, Germany). To purify blood GR1+CD11b+ cells, monocytes from whole blood were negatively selected with murine monocytes spin sep kit (Stem Cell Technologies, Vancouver, BC, Canada) following the manufacturer’s instructions.

### 4.3. Flow Cytometry Analysis

Single cell suspensions or purified CD11b+ cells were washed with macs buffer (miltenyi biotech) and labelled for 30 min on ice with fluorochrome-conjugated antibodies: anti-GR1, CD11b, F4/80, MHCII (eBioscience, San Diego, CA, USA), CD86 (Becton-Dickinson, Le Pont de Claix, France), CD206 (Serotec, Colmar, France), and isotype-matched IgG controls. After washing, stained cells were analyzed on an ADPCyan flow cytometer (Beckman-Coulter, Villepinte, France). Data analyses were performed with Summit software (Beckman). To isolate different populations of tumor infiltrating-CD11b+ cells, pre-sorted CD11b+ cells were stained with APC-anti GR1 and Alexa 488-anti F4/80 antibodies (eBioscience). Different GR1+/− F4/80+/− populations were isolated on the FACS sorter FACSaria II (BD) with the strategy of gating illustrated in Figure 3.

### 4.4. Immunosuppression Assay

CD4+ T cells were purified from normal mouse spleen by using mouse CD4+ T cell kit (Miltenyi Biotec), labelled with CFSE (10 µM, 10 min at 37 °C) (Life Technologies, Saint Aubin, France) and 10^5^ cells were added to flat-bottom 96-well plates (BD) and stimulated with anti CD3/CD28 beads (Life Technologies, Saint Aubin, France). GR1CD11b cells were purified either from the spleen of normal or from 4T1-bearing mice and added at different ratios with purified CD4+ T cells. After 4 days, the cocultures were stopped and analyzed on an ADPCyan flow cytometer (Beckman). Data analysis was performed with Summit software (Beckman). The percentage of suppression of T cell proliferation with 4T1 derived GR1CD11b cells was calculated as follows: [1 − (proliferation with 4T1 GR1CD11b cells/proliferation with normal GR1CD11b cells)] × 100.

### 4.5. *In Vitro* Proliferation and Differentiation of GR1CD11b Cells

CFSE-labelled purified GR1CD11b cells obtained from spleen or blood of normal mice were cultured in RPMI supplemented with 10% FCS and 1% Glutamine. The cells were cultured alone, or supplemented with recombinant murine MCSF (100 ng/mL; R&D Systems, Minneapolis, MN, USA) or with conditioned media obtained from 4T1 cell culture supernatant harvested after 48 h. CFSE labeling was analyzed after 48–72 h on a ADPCyan flow cytometer (Beckman). Data analyses were performed with Summit software (Beckman). Non-labeled GR1CD11b cells were used to quantify ATP content in the same experiment setting. The protocol to monitor ATP incorporation was described in Favier *et al*. [46]. For differentiation studies, GR1CD11b cells were restimulated after 3–4 days and harvested for analysis at Day 7. To study the differentiation status of GR1CD11b cells in TAMs, cells were labeled with APC-anti GR1 and Alexa 488- anti F4/80 antibodies (eBioscience) and analyzed on an ADPCyan flow cytometer (Beckman).

### 4.6. M1/M2 Polarization in a Murine Model

Purified GR1CD11b cells were differentiated into macrophages during six days in RPMI containing 10% FCS, 1% glutamine, in the presence of 100 ng/mL of recombinant murine MCSF (R&D). At Day 6, macrophages were polarized during 24 h towards the M1 phenotype with murine recombinant IFNγ (20 ng/mL; R&D) + LPS ultrapure K12 (1 ng/mL; Cayla Invivogen, Toulouse, France) treatment and towards M2 profile with murine recombinant IL-4 (20 ng/mL; R&D) or with 4T1 conditioned medium.

### 4.7. Transcriptomic Analysis

Transcriptomic analysis was performed on ARN prepared from sorted splenic GR1CD11b cells and sorted tumor infiltrating GR1^High^/F4/80^−^, GR1^Int^/F4/80^High^ and GR1^Low^/F4/80^Low^ isolated on FACS sorter FACSaria II (BD).

Total RNA was prepared using RNeasy micro kit (Qiagen, Hilden, Germany) including DNAse treatment step (Rnase-free DNase kit, Qiagen). RNA was reverse-transcribed using the High Capacity cDNA Archive kit (Applied Biosystems, Life Technologies). Polymerase Chain Reactions were performed using commercial TaqMan assays (Applied Biosystems), Taqman Universal Master Mix (4304437, Applied Biosystems) and an ABI7900HT machine (Applied Biosystems). Cycles were as follows: 15 s at 95 °C and 1 min at 60 °C, 40 times. Ct (cycle threshold) values were determined using RQ manager 1.2 software (Applied Biosystems) with automatic baseline and threshold, and RQ min/max confidence = 95%. Ct is the cycle at which amplification signal is significantly above the background. Data analysis was performed using RealTime Statminer™ software (Integromics, Madrid, Espagne). A parametric test was performed (Limma option) with Benjamini-Hochberg False Discovery Rate p-value correction to assess significancy of expression differences between myeloid cell subpopulations. Abundance relative to 18S were calculated as RelA = 2 power (−DCt) × 1E9, where DCt is the difference between Ct of gene of interest and Ct of 18S. 

## 5. Conclusions

This work strongly suggest that in addition to its role on lymphatic and endothelial vasculature, the specific VEGFR-3 inhibitor SAR131675 has an anti tumor effect by acting on different mechanisms through which key players of inflammatory response orchestrate tumor progress, confirming that VEGFR-3 axis is an interesting therapeutic target for cancer treatment. Moreover these data highlight the fact that the specific inhibition of VEGFR3 tyrosine kinase is more suitable than the blockade of multikinase pathways pointing out the interest of a new class of specific kinase inhibitors for cancer treatment.

## Supplementary Files

Supplementary File 1 Supplementary Material (PDF, 473 KB)

## Abbreviations

VEGFR: Vascular Endothelial Growth Factor Receptor
TAMs: Tumor Associated Macrophages
MDSC: Myeloid Derived Suppressive Cells

## References

[1] Almand B., Clark J.I., Nikitina E., van Beynen J., English N.R., Knight S.C., Carbone D.P., Gabrilovich D.I. (2001). Increased production of immature myeloid cells in cancer patients: A mechanism of immunosuppression in cancer. J. Immunol..

[2] Pak A.S., Wright M.A., Matthews J.P., Collins S.L., Petruzzelli G.J., Young M.R. (1995). Mechanisms of immune suppression in patients with head and neck cancer: Presence of CD34(+) cells which suppress immune functions within cancers that secrete granulocyte-macrophage colony-stimulating factor. Clin. Cancer Res..

[3] Zea A.H., Rodriguez P.C., Atkins M.B., Hernandez C., Signoretti S., Zabaleta J., McDermott D., Quiceno D., Youmans A., O’Neill A. (2005). Arginase-producing myeloid suppressor cells in renal cell carcinoma patients: A mechanism of tumor evasion. Cancer Res..

[4] Baniyash M. (2006). Chronic inflammation, immunosuppression and cancer: New insights and outlook. Semin. Cancer Biol..

[5] Ostrand-Rosenberg S., Sinha P. (2009). Myeloid-derived suppressor cells: Linking inflammation and cancer. J. Immunol..

[6] Gabrilovich D.I., Nagaraj S. (2009). Myeloid-derived suppressor cells as regulators of the immune system. Nat. Rev. Immunol..

[7] Movahedi K., Guilliams M., van den Bossche J., van den Bergh R., Gysemans C., Beschin A., de Baetselier P., van Ginderachter J.A. (2008). Identification of discrete tumor-induced myeloid-derived suppressor cell subpopulations with distinct T cell-suppressive activity. Blood.

[8] Youn J.I., Nagaraj S., Collazo M., Gabrilovich D.I. (2008). Subsets of myeloid-derived suppressor cells in tumor-bearing mice. J. Immunol..

[9] Kusmartsev S., Su Z., Heiser A., Dannull J., Eruslanov E., Kubler H., Yancey D., Dahm P., Vieweg J. (2008). Reversal of myeloid cell-mediated immunosuppression in patients with metastatic renal cell carcinoma. Clin. Cancer Res..

[10] Diaz-Montero C.M., Salem M.L., Nishimura M.I., Garrett-Mayer E., Cole D.J., Montero A.J. (2009). Increased circulating myeloid-derived suppressor cells correlate with clinical cancer stage, metastatic tumor burden, and doxorubicin-cyclophosphamide chemotherapy. Cancer Immunol. Immunother..

[11] Liu C.Y., Wang Y.M., Wang C.L., Feng P.H., Ko H.W., Liu Y.H., Wu Y.C., Chu Y., Chung F.T., Kuo C.H. (2010). Population alterations of L-arginase- and inducible nitric oxide synthase-expressed CD11b+/CD14(−)/CD15+/CD33+ myeloid-derived suppressor cells and CD8+ T lymphocytes in patients with advanced-stage non-small cell lung cancer. J. Cancer Res. Clin. Oncol..

[12] Wang L., Chang E.W., Wong S.C., Ong S.M., Chong D.Q., Ling K.L. (2013). Increased myeloid-derived suppressor cells in gastric cancer correlate with cancer stage and plasma S100A8/A9 proinflammatory proteins. J. Immunol..

[13] Martinez F.O., Helming L., Gordon S. (2009). Alternative activation of macrophages: An immunologic functional perspective. Annu. Rev. Immunol..

[14] Sica A., Schioppa T., Mantovani A., Allavena P. (2006). Tumour-associated macrophages are a distinct M2 polarised population promoting tumour progression: Potential targets of anti-cancer therapy. Eur. J. Cancer.

[15] Murdoch C., Muthana M., Coffelt S.B., Lewis C.E. (2008). The role of myeloid cells in the promotion of tumour angiogenesis. Nat. Rev. Cancer.

[16] Lin C.Y., Lin C.J., Chen K.H., Wu J.C., Huang S.H., Wang S.M. (2006). Macrophage activation increases the invasive properties of hepatoma cells by destabilization of the adherens junction. FEBS Lett..

[17] Pollard J.W. (2004). Tumour-educated macrophages promote tumour progression and metastasis. Nat. Rev. Cancer.

[18] Ryder M., Ghossein R.A., Ricarte-Filho J.C., Knauf J.A., Fagin J.A. (2008). Increased density of tumor-associated macrophages is associated with decreased survival in advanced thyroid cancer. Endocr. Relat. Cancer.

[19] Shabo I., Stal O., Olsson H., Dore S., Svanvik J. (2008). Breast cancer expression of CD163, a macrophage scavenger receptor, is related to early distant recurrence and reduced patient survival. Int. J. Cancer.

[20] Fridlender Z.G., Sun J., Mishalian I., Singhal S., Cheng G., Kapoor V., Horng W., Fridlender G., Bayuh R., Worthen G.S. (2012). Transcriptomic analysis comparing tumor-associated neutrophils with granulocytic myeloid-derived suppressor cells and normal neutrophils. PLoS One.

[21] Gabrilovich D.I., Ostrand-Rosenberg S., Bronte V. (2012). Coordinated regulation of myeloid cells by tumours. Nat. Rev. Immunol..

[22] Kusmartsev S., Gabrilovich D.I. (2005). STAT1 signaling regulates tumor-associated macrophage-mediated T cell deletion. J. Immunol..

[23] Alam A., Blanc I., Gueguen-Dorbes G., Duclos O., Bonnin J., Barron P., Laplace M.C., Morin G., Gaujarengues F., Dol F. (2012). SAR131675, a potent and selective VEGFR-3-TK inhibitor with antilymphangiogenic, antitumoral, and antimetastatic activities. Mol. Cancer Ther..

[24] DuPre S.A., Hunter K.W. (2007). Murine mammary carcinoma 4T1 induces a leukemoid reaction with splenomegaly: Association with tumor-derived growth factors. Exp. Mol. Pathol..

[25] Pulaski B.A., Ostrand-Rosenberg S. (2001). Mouse 4T1 breast tumor model. Curr. Protoc. Immunol..

[26] Koebel C.M., Vermi W., Swann J.B., Zerafa N., Rodig S.J., Old L.J., Smyth M.J., Schreiber R.D. (2007). Adaptive immunity maintains occult cancer in an equilibrium state. Nature.

[27] Sinha P., Clements V.K., Ostrand-Rosenberg S. (2005). Interleukin-13-regulated M2 macrophages in combination with myeloid suppressor cells block immune surveillance against metastasis. Cancer Res..

[28] Shojaei F., Wu X., Malik A.K., Zhong C., Baldwin M.E., Schanz S., Fuh G., Gerber H.P., Ferrara N. (2007). Tumor refractoriness to anti-VEGF treatment is mediated by CD11b+Gr1+ myeloid cells. Nat. Biotechnol..

[29] Movahedi K., Laoui D., Gysemans C., Baeten M., Stange G., van den Bossche J., Mack M., Pipeleers D., In’t Veld P., de Baetselier P. (2010). Different tumor microenvironments contain functionally distinct subsets of macrophages derived from Ly6C(high) monocytes. Cancer Res..

[30] Loercher A.E., Nash M.A., Kavanagh J.J., Platsoucas C.D., Freedman R.S. (1999). Identification of an IL-10-producing HLA-DR-negative monocyte subset in the malignant ascites of patients with ovarian carcinoma that inhibits cytokine protein expression and proliferation of autologous T cells. J. Immunol..

[31] Schartner J.M., Hagar A.R., van Handel M., Zhang L., Nadkarni N., Badie B. (2005). Impaired capacity for upregulation of MHC class II in tumor-associated microglia. Glia.

[32] Fridlender Z.G., Albelda S.M. (2012). Tumor-associated neutrophils: Friend or foe?. Carcinogenesis.

[33] Gu Y., Zhao W., Meng F., Qu B., Zhu X., Sun Y., Shu Y., Xu Q. (2010). Sunitinib impairs the proliferation and function of human peripheral T cell and prevents T-cell-mediated immune response in mice. Clin. Immunol..

[34] Zhao W., Gu Y.H., Song R., Qu B.Q., Xu Q. (2008). Sorafenib inhibits activation of human peripheral blood T cells by targeting LCK phosphorylation. Leukemia.

[35] Van Dongen M., Savage N.D., Jordanova E.S., Briaire-de Bruijn I.H., Walburg K.V., Ottenhoff T.H., Hogendoorn P.C., van der Burg S.H., Gelderblom H., van Hall T. (2010). Anti-inflammatory M2 type macrophages characterize metastasized and tyrosine kinase inhibitor-treated gastrointestinal stromal tumors. Int. J. Cancer.

[36] Zhang W., Zhu X.D., Sun H.C., Xiong Y.Q., Zhuang P.Y., Xu H.X., Kong L.Q., Wang L., Wu W.Z., Tang Z.Y. (2010). Depletion of tumor-associated macrophages enhances the effect of sorafenib in metastatic liver cancer models by antimetastatic and antiangiogenic effects. Clin. Cancer Res..

[37] Baluk P., Tammela T., Ator E., Lyubynska N., Achen M.G., Hicklin D.J., Jeltsch M., Petrova T.V., Pytowski B., Stacker S.A. (2005). Pathogenesis of persistent lymphatic vessel hyperplasia in chronic airway inflammation. J. Clin. Investig..

[38] Cursiefen C., Chen L., Borges L.P., Jackson D., Cao J., Radziejewski C., D’Amore P.A., Dana M.R., Wiegand S.J., Streilein J.W. (2004). VEGF-A stimulates lymphangiogenesis and hemangiogenesis in inflammatory neovascularization via macrophage recruitment. J. Clin. Investig..

[39] Hamrah P., Chen L., Zhang Q., Dana M.R. (2003). Novel expression of vascular endothelial growth factor receptor (VEGFR)-3 and VEGF-C on corneal dendritic cells. Am. J. Pathol..

[40] Maruyama K., Ii M., Cursiefen C., Jackson D.G., Keino H., Tomita M., van Rooijen N., Takenaka H., D’Amore P.A., Stein-Streilein J. (2005). Inflammation-induced lymphangiogenesis in the cornea arises from CD11b-positive macrophages. J. Clin. Investig..

[41] Shin Y.J., Choi J.S., Choi J.Y., Hou Y., Cha J.H., Chun M.H., Lee M.Y. (2010). Induction of vascular endothelial growth factor receptor-3 mRNA in glial cells following focal cerebral ischemia in rats. J. Neuroimmunol..

[42] Schoppmann S.F., Birner P., Stockl J., Kalt R., Ullrich R., Caucig C., Kriehuber E., Nagy K., Alitalo K., Kerjaschki D. (2002). Tumor-associated macrophages express lymphatic endothelial growth factors and are related to peritumoral lymphangiogenesis. Am. J. Pathol..

[43] Yang H., Kim C., Kim M.J., Schwendener R.A., Alitalo K., Heston W., Kim I., Kim W.J., Koh G.Y. (2011). Soluble vascular endothelial growth factor receptor-3 suppresses lymphangiogenesis and lymphatic metastasis in bladder cancer. Mol. Cancer.

[44] Alitalo A.K., Proulx S.T., Karaman S., Aebischer D., Martino S., Jost M., Schneider N., Bry M., Detmar M. (2013). VEGF-C and VEGF-D blockade inhibits inflammatory skin carcinogenesis. Cancer Res..

[45] Bogos K., Renyi-Vamos F., Dobos J., Kenessey I., Tovari J., Timar J., Strausz J., Ostoros G., Klepetko W., Ankersmit H.J. (2009). High VEGFR-3-positive circulating lymphatic/vascular endothelial progenitor cell level is associated with poor prognosis in human small cell lung cancer. Clin. Cancer Res..

[46] Favier B., Alam A., Barron P., Bonnin J., Laboudie P., Fons P., Mandron M., Herault J.P., Neufeld G., Savi P. (2006). Neuropilin-2 interacts with VEGFR-2 and VEGFR-3 and promotes human endothelial cell survival and migration. Blood.

