# Supplementary material for: Specific Inhibition of the VEGFR-3 Tyrosine Kinase by SAR131675 Reduces Peripheral and Tumor Associated Immunosuppressive Myeloid Cells
Source: Cancers (Basel). 2014 Feb 28;6(1):472–90. doi: 10.3390/cancers6010472 (PMC3980599; doi:10.3390/cancers6010472)
Supplement: Supplementary File 1 — Supplementary Material (PDF, 473 KB) [file cancers-06-00472-s001.pdf]

## Supplementary Material

**Figure S1.** blood GR1CD11b cell characterization in the 4T1 model and effect of SAR 131675. (**A**) Dot plots of blood GR1CD11b cells from control (**a**) and 4T1-bearing mice (**b**) and kinetics of blood GR1CD11b cell proportion during tumor progression (**c**). (**B**) GR1CD11b cells were purified from control and 4T1-derived bone marrow, blood and spleen, by CD11b immunomagnetic sorting. Purified GR1CD11b cells were stained with fluorochrome-coupled anti-MHCII, CD86, F4/80 antibodies. Summary table of phenotypic characterization between control and 4T1 purified GR1CD11b cells from bone marrow, blood and spleen is shown. (**C**) Proportion of splenic CD4+ T cells and GR1CD11b from control and 4T1 mice. Physiologic and physiopathological ratio (GR1CD11b cells/CD4 T cells) were calculated. (**D**) Effect of SAR131675 on the proportion of 4T1 circulating GR1CD11b cells. Blood derived GR1CD11b cells from tumor free mice were used as control.

A

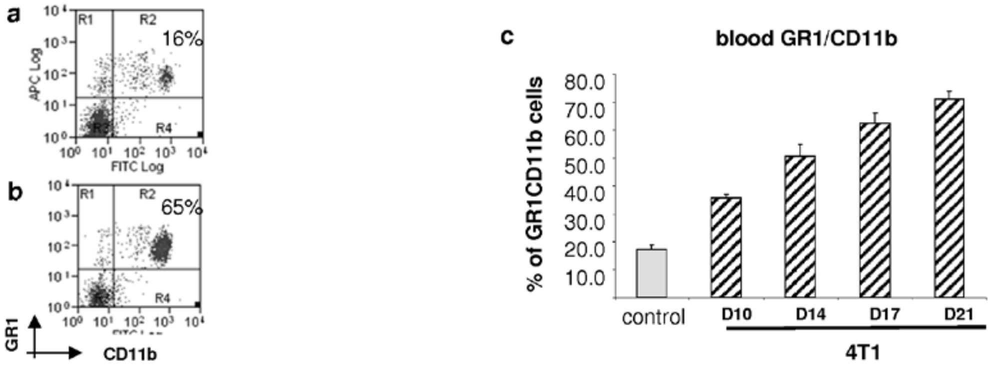


B

| Markers | Bone marrow GR1CD11b | | Blood GR1CD11b | | Spleen GR1CD11b | |
|---|---|---|---|---|---|---|
| | ctl | 4T1 | ctl | 4T1 | ctl | 4T1 |
| GR1 | +++ | +++ | +++ | +++ | +++ | +++ |
| CD11b | + | + | + | + | + | + |
| F4/80 | - | - | - | - | - | - |
| MHC-II | -/Low | -/Low | -/Low | -/Low | -/Low | -/Low |
| CD86 | - | - | - | - | - | - |

C

| Proportion of splenic cells | Control mice | 4T1 bearing mice |
|---|---|---|
| % CD4 | 45 % | 5 % |
| % GR1 CD11b | 5-10 % | 50 % |
| GR1CD11b / CD4 ratio | 0.1-0.2 | 10 |

D

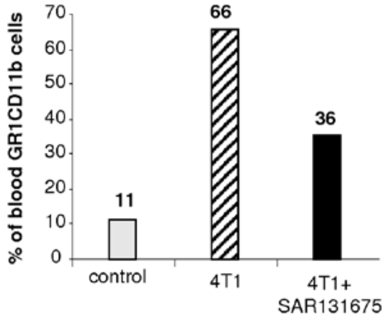

**Figure S2.** (**A**) Cytometric analysis of CD115 and IL4R on CD11b cells isolated from spleen of 4T1 mice. (**B**) Analysis of MCSF expression in 4T1 supernatant (SN) and plasma of WT or 4T1 bearing mice, using a protoarray approach. IL13 has been evaluated by ELISA from 4T1 tumor lysate and skin lysate has been used as control. VEGFC has been quantified by ELISA from 4T1 tumor lysate diluted 1/100 (mean ± SD of 5 tumors is shown) and from supernatant of 4T1 cells obtained 24 h after cell culture of 80% confluent cells. The supernatant was used pure for the ELISA. (**C**) Effect of SAR131675 and sunitinib on the autophosphorylation of MCSF1R in HEK cells. Results show that up 1000 nM, in opposite to sunitib, SAR131675 has no effect on MCSFR autophosphorylation.

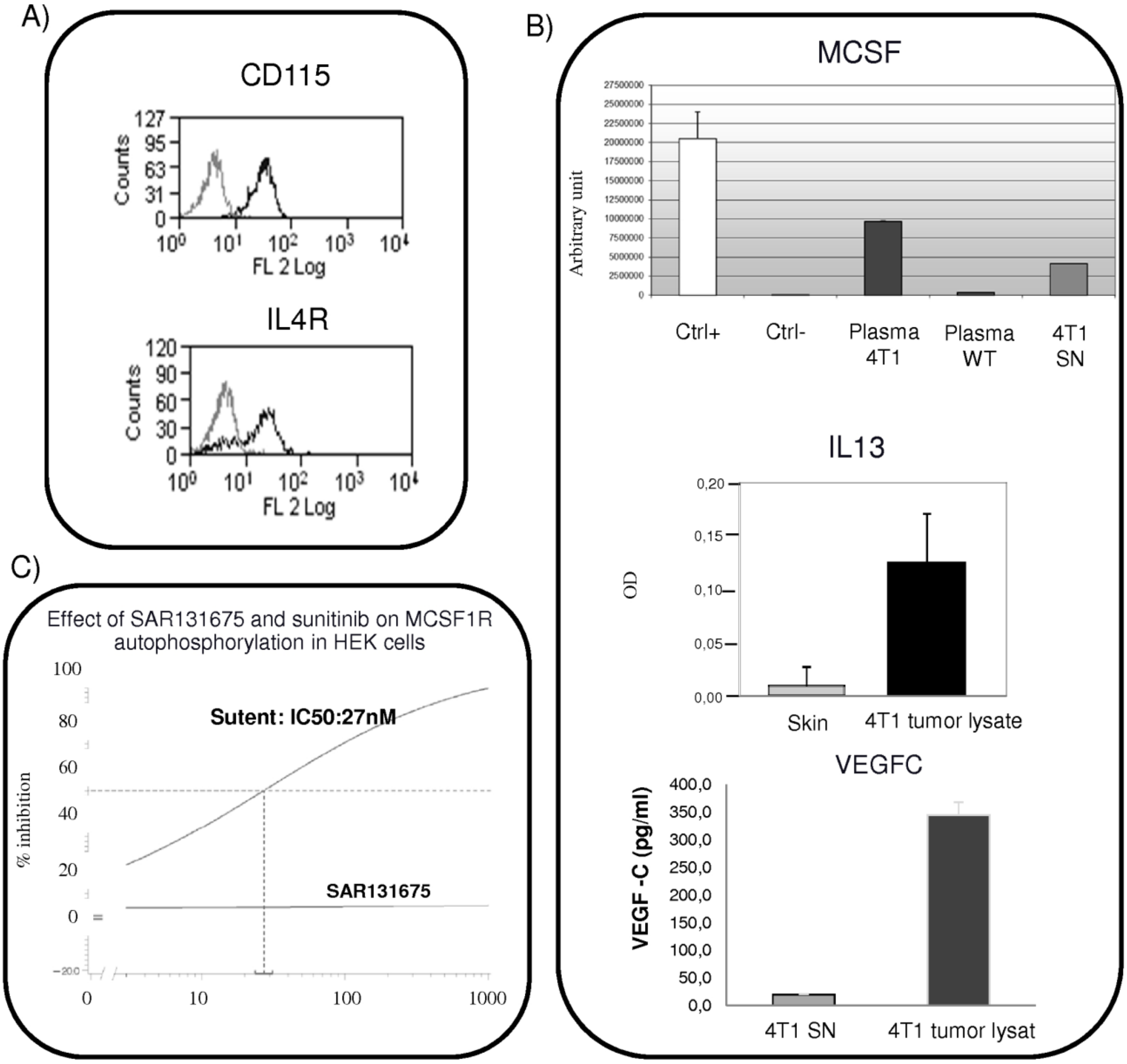

**Figure S3.** Effect of SAR131675 on tumor induced GR1CD11b cells and TAMs. Tumor can induce GR1CD11b cell production in bone marrow via soluble factors causing an increase of GR1CD11b cells in blood and spleen of tumor-bearing mice. These GR1CD11b cells inhibit T cell proliferation in peripheral lymphoid organs. GR1CD11b cells could migrate into the tumor and differentiate into F4/80+ macrophages under influence of tumor derived factors such as MCSF. Two distinct macrophage populations are infiltrated in the tumor; "immuno-incompetent-M2" (GR1$^{Low}$/F4/80$^{High}$/MHCII$^{Low}$/CD86−) and "immunocompetent-M1" macrophages (GR1$^{Low}$/F4/80$^{Low}$/MHCII$^{High}$/CD86+) cells. The specific VEGFR3 inhibitor SAR131675 inhibits angiogenesis and lymphangiogenesis. Here we showed that SAR131675 reduces tumor induced blood and splenic GR1CD11b cells (MDSCs) in the periphery of tumor and reduces the number of M2-promoting tumor macrophages while increasing the M1 macrophages in the tumor microenvironment. These schema depicts how SAR131675 could act on multiple key players to reduce tumor growth and metastasis.

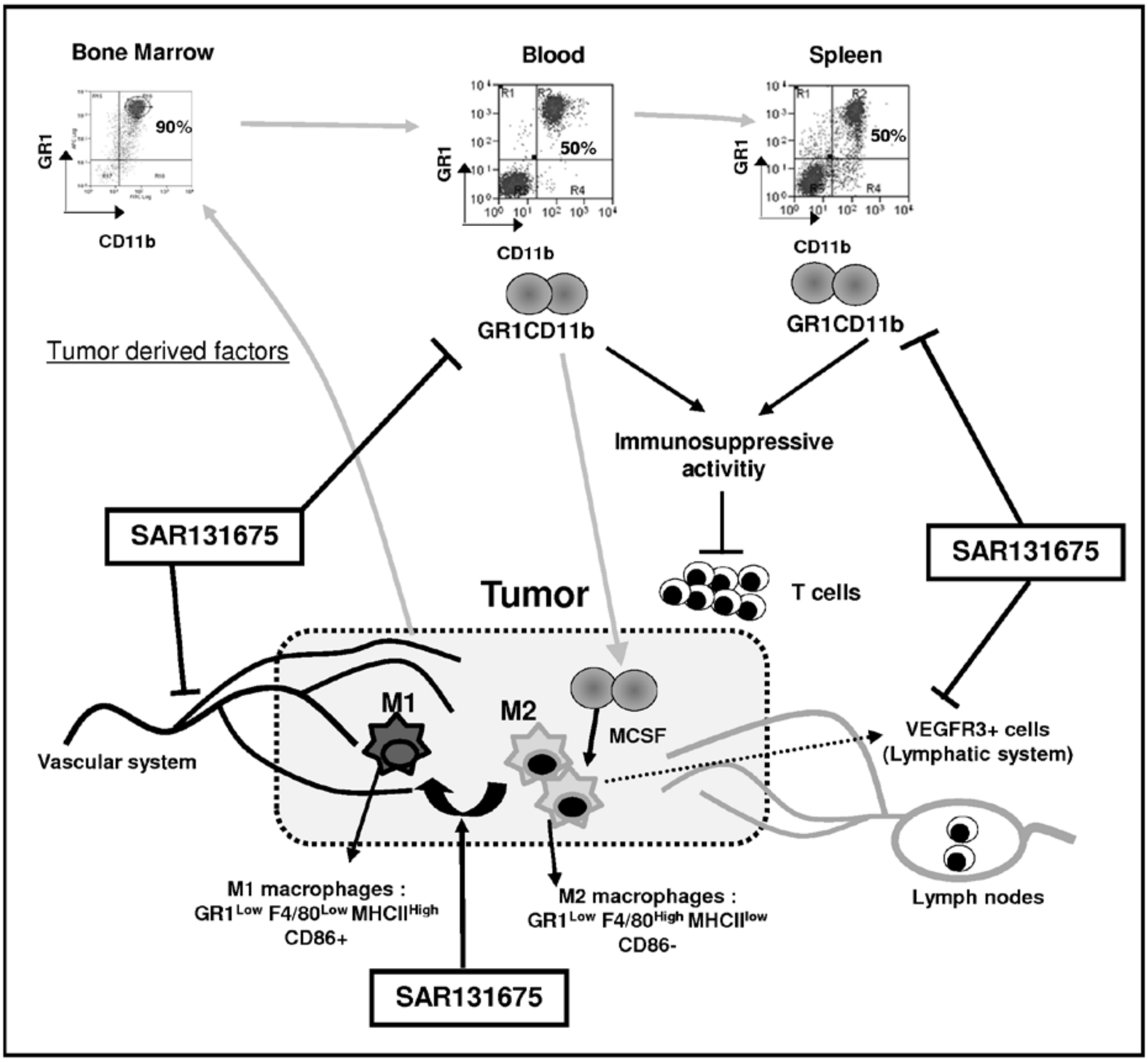


© 2014 by the authors; licensee MDPI, Basel, Switzerland. This article is an open access article distributed under the terms and conditions of the Creative Commons Attribution license (http://creativecommons.org/licenses/by/3.0/).
